# The High Content of Quercetin and Catechin in Airen Grape Juice Supports Its Application in Functional Food Production

**DOI:** 10.3390/foods10071532

**Published:** 2021-07-02

**Authors:** Daniel J. García-Martínez, María Arroyo-Hernández, María Posada-Ayala, Cruz Santos

**Affiliations:** Bioscience Research Institute, Faculty of Experimental Sciences, Universidad Francisco de Vitoria, Ctra. M-515, Pozuelo-Majadahonda Km 1.800, Pozuelo de Alarcón, 28223 Madrid, Spain; d.garciamartinez@ufv.es (D.J.G.-M.); m.arroyo.prof@ufv.es (M.A.-H.)

**Keywords:** grape juice, polyphenols, antioxidants, *Vitis vinifera* var. Airen

## Abstract

Ensuring healthy lives and well-being constitutes one of the Sustainable Development Goals of the UN 2030 agenda. Consequently, research into how natural products may promote health is essential for the new generation of nutraceuticals and functional foods that are in high demand today. Grape juice is a natural foodstuff composed of water, sugars, minerals, vitamins and a wide array of polyphenols. Polyphenols are bioactive compounds of great interest due to their antioxidant properties and benefits to health, supporting antimicrobial, anti-aging, and anticarcinogenic activity. The majority of grape juice produced in the world is used for the production of wine, although a small part is used in the food industry, mainly in baby food and sports drinks. The aim of this work is to determine the polyphenol content in the natural and concentrated juice of Airen grapes, the main white grape variety produced in Spain. For this, fresh juices from five grape varietals (Airen, Sauvignon Blanc, Gewürztraminer, Verdejo and Tempranillo) and concentrated Airen juice were analyzed and compared. Results showed similar contents of phenolic acids and stilbenes in all grape varietals studied, although the Airen variety demonstrated a higher concentration of two flavonoids: quercetin and catechin. It can be concluded that the grape juice concentration process negatively affects the stability of these compounds, causing a reduction in the polyphenol content that ranges between 54–71%, with the exception of quercetin and catechin.

## 1. Introduction

Grape juice is a product derived from grape berries. Grapes—a popular staple in the Mediterranean diet—comprise water and sugars, glucose and fructose, together with small quantities of minerals, vitamins and other organic compounds known as phytochemicals. Phenolic compounds belong to this group of organic molecules present in plants and fruits that show interesting properties related to human health [1]. The antioxidant capacity of these compounds has been extensively demonstrated, particularly as they relate to their anti-aging, anti-inflammatory, cardioprotective and immunomodulating properties [2,3,4,5,6]. Moreover, there is evidence suggesting that the antimicrobial and anticarcinogenic properties of specific polyphenol compounds are associated with the flavonoid and stilbene families [7]. All this evidence has promoted greater interest in these bioactive molecules regarding their use as nutraceuticals in order to improve the quality of foods, particularly functional foods tailored for children, sportsmen, and people suffering from different diseases.

Spain has a great tradition in the culture of vine and wine production. The Airen variety of *Vitis vinifera* is the main white grape cultured (occupying 215,546 hectares) and constitutes 23% of the total vineyard area of the country and 50% of the white varieties [8]. Other white grapes cultured in Spain, such as Verdejo, Gewürztraminer and Sauvignon Blanc, constitute only 2% of the cultured vine surface. Castilla-La Mancha is the Spanish region with the highest vineyard area of the Airen variety, which is mainly utilized for wine production. However, approximately 20% of the Airen grapes grown are used in the production of concentrated grape juice, a product required for the chaptalization process in producing wine, as well as in the food industry for the production of baby foods and drinks, including sports beverages.

The inclusion of grape juice in drinks and foods is valued because of the polyphenol content and its beneficial properties for promoting health and preventing the development of diseases [9,10,11]. The amount and type of phenolic compounds present in grape juice depend on the grape variety, the climate, the viticulture conditions, and the process of juice obtention. To date, these compounds have not been extensively studied. The majority of the polyphenols are located in the seeds and skins of grape berries, whereas the pulp contains less of these compounds [3,12,13]. The skin and seeds possess complex polyphenols responsible for bitter and astringent flavors, characteristics not so greatly appreciated in food products. Grape juice obtained from the pulp of specific grape species is a natural product with bioactive molecules; this juice is in high demand for use in nonalcoholic drinks such as juices, drinks for babies, restorative drinks and energy shakes [14,15].

Past studies have shown that consumption of polyphenol-rich foods reduces the risk of oxidative stress-induced disease, due to their antioxidant properties, lowering the accumulation of intracellular reactive oxygen species (ROS) that are important molecules in the development of neurodegenerative, cardiovascular, and cancerous diseases [16,17]. There are in vivo studies and clinical trials using grape polyphenols that have shown their beneficial effects in the treatment of cancer [18,19,20] and cardiovascular diseases [21,22]. Moreover, research examining specific polyphenols such as resveratrol has shown them to interfere with numerous metabolic pathways related to the progression of some types of cancer and coronary heart disease [23,24]. Other polyphenols also present in grapes, such as quercetin and its derivatives, have been involved in the management of inflammation and pain [25], and have shown interesting anticarcinogenic and proapoptotic properties when used in the treatment of certain cancer types [19,26,27].

In recent years, numerous studies have characterized the polyphenol content in wines. This research has shown that the amount of these compounds in red wines is significantly higher than in white wines because of the grape variety and the technological processes involved in their production [28,29]. However, recent epidemiological and in vitro studies suggest that white wine could have similar health benefits when compared to red wine [30,31,32,33,34]. Moreover, it has been demonstrated that the antioxidant capacity of polyphenols present in white grape varieties is not negligible, which adds value to any product derived from such varieties, included grape juice [35]. A recent study has shown that the bioactive molecules present in both grape juice and wine are responsible for health benefits when included in the diet. Nevertheless, the alcohol present in wines is not recommended for children, elderly individuals, and people with different pathologies [36]. Moreover, it has been reported that consumption of grape juice has similar antioxidant effects to wine, despite the higher quantity of polyphenols present in wine [37]. There are several studies showing the positive effects of grape juice consumption for human health, including reductions in body mass index, glycemia, peroxidation of plasma lipids, blood pressure and total cholesterol, as well as increases in serum antioxidant capacity and the plasmatic levels of HDL-c and apolipoprotein B [37,38,39,40,41,42,43,44]. These outcomes continue to fuel interest in better understanding the polyphenol composition of grape juice and the beneficial effects on health when included in the daily diet [12,14,45].

Most phenolic compounds in white grapes belong to the non-flavonoid group, comprising mainly phenolic acids (gallic, protocatechuic, syringic, vanillic and ellagic acids), and flavonoids, including the flavanols (catechin, epicatechin, procyanidins and higher oligomers) and flavonols (quercetin and other five aglycones, mainly as glycosides). All these phenolics have been reported to have cardioprotective, neuroprotective, anticancer, antioxidant, anti-inflammatory, and antimicrobial properties [3,4,14,46], thus supporting the present study’s aim to determine the polyphenol composition of Airen grape juice, a product in high demand in the food industry. The main objective of this work was to characterize the polyphenol content in the natural and concentrated Airen grape juices produced in the Castilla-La Mancha Spanish region. For this purpose, grape juice samples from four white grape varieties (Airen, Sauvignon Blanc, Verdejo and Gewürztraminer) and the red variety Tempranillo were analyzed.

## 2. Materials and Methods

### 2.1. Chemicals and Reagents

The solvents used for polyphenol extraction and liquid chromatographic mass spectrometry (LC-MS/MS) analysis, methanol, acetonitrile and formic acid, were purchased from Merck (Darmstadt, Germany). 2,2-diphenyl-1picrylhydrazyl (DPPH), used to determine the antioxidant capacity, was purchased from Thermo Fisher (Kandel, Germany). The polyphenols used as standards, aminobenzoic acid, acetylsalicylic acid, caffeic acid, chlorogenic acid, ellagic acid, gallic acid, p-coumaric acid, protocatechuic acid, salicylic acid, trans-ferulic acid, vanillic acid, apigenin, epicatechin, aesculetin, catechin hydrate, isorhamnetin, kaempferol, luteolin, polydatin, quercetin, resveratrol, rutin, syringaldehyde and viniferin, were purchased from Sigma-Aldrich (Madrid, Spain). The Mili-Q water used in all the solutions was purified with the Merck Millipore Milli-Q™ Reference Ultrapure Water Purification System model Z00QSVC01 (Darmstadt, Germany).

### 2.2. Grape Juice Samples and Polyphenol Extraction

Fresh juices from four different white grape varieties of *Vitis vinifera* (Airen, Sauvignon Blanc, Gewürztraminer and Verdejo), and the red variety Tempranillo, were analyzed. All the vineyards were located in Castilla-La Mancha, Spain, and the juice samples were supplied by the winery Vinicola de Tomelloso (Tomelloso, Spain) during the 2017 and 2018 harvests. Once the quality control was carried out by the winery’s oenologist, the samples were collected and frozen at −20 °C until their laboratory processing.

The concentrated grape juice samples were obtained from the company Mostos Españoles S.A., located in Tomelloso, Spain. The concentration process consisted of heating the grape juice at 95 °C to evaporate water, increasing the concentration of sugars from 19 to 65 Brix degrees (grams of sugar per 100 mL of juice). To obtain discolored concentrated grape juice, a filtration step through a nitrocellulose tubular membrane of 0.45-micrometer pore diameter (Permeare, Padova, Italy), was performed before the concentration. This process allowed the removal of compounds responsible for the color, in addition to minerals, ions such as iron, magnesium, calcium or potassium, and possibly other bioactive molecules present in the juice [9,15]. Industrial samples were collected at three stages of the concentration process in both normal and decolorized concentrated juice (NCJ and DCJ, respectively): initial at 19 °Bx (NCJ_19_/DCJ_19_), intermediate at 30 °Bx (NCJ_30_/DCJ_30_), and final product at 65 °Bx (NCJ_65_/DCJ_65_). The concentrated juice contains 3.5 times more sugar than fresh grape juice.

The extraction of polyphenols was carried out following the procedure described below, based on those previously described for the extraction of these compounds from grape bunches, skins, and seeds [10,11,47]. The method was optimized with standard polyphenols that are commercially available. These compounds were extracted with different solvents: methanol, ethanol, and acetone, all of them 100% and 50% diluted with Mili-Q water. After that, the polyphenols were quantified by spectrophotometric measurement at 280 nm, showing that extraction with pure methanol resulted in no significant molecular loss.

Fresh and concentrated grape juice samples of 0.2 mL were lyophilized, and the solid dry matrix was used as the substrate for the extraction. The polyphenol extraction was performed by adding 1.0 mL of methanol to the solid matrix (ratio 1:5 *v/v*) and the extraction was done over 2 h at 4 °C with gentle rotatory mixing. The samples were then centrifuged at 13,000 rpm and 4 °C, and the supernatant was recovered and filtrated using a 0.45 µM polytetrafluoroethylene membrane filter (hydrophilic PTFE) purchased by Merck (Darmstadt, Germany). The obtained polyphenol extracts were frozen at −80 °C until the analysis by LC-MS/MS. Twelve different extracts of each grape juice sample were analyzed in this study.

### 2.3. Estimation of Total Polyphenols

The quantity of total polyphenols in the extracts and the grape juice samples was estimated by spectrophotometry at 280 nm using gallic acid at known concentrations (ranging between 2 and 20 mg/L), as a reference. A calibration curve with gallic acid (y = 0.0179x − 0.0376; *R*^2^ = 0.9998) was used to determine the polyphenol content in mg/L gallic acid equivalents (GAE).

### 2.4. DPPH Radical Scavenging Assay

Free-radical scavenging activity of the grape juice samples and the polyphenol extracts was determined following the procedure described by Brand-Williams [48] with some modifications [49]. The oxidative compound DPPH was used as a substrate, and the IC_50_ values were calculated by expressing the concentration (mg/L) of polyphenol (or extract) that scavenges the DPPH radical by 50%. The assays were performed in 96-well plates (Nunc Delta Surface) with 200 µL of DPPH 60 µM dissolved in methanol, with variable amounts of grape juice or polyphenol extracts (0–20 µL). The mixtures were incubated for 30 min at room temperature in the dark, and the reaction was followed by measurements of the absorbance at 562 nm in a TECAN Sunrise spectrophotometer (Zurich, Switzerland). Gallic acid was included in the assay as a control. The lowest IC_50_ values indicate the highest antioxidant capacity of the sample.

### 2.5. LC-MS/MS Analysis

Polyphenol extracts were analyzed on QTrap 4500 mass spectrometry system (Sciex, Darmstadt, Germany) equipped with a Turbo V electrospray ionization source. Data was acquired using Analyst software 1.6 (Sciex, Darmstadt, Germany). Mass spectrometry operation was coupled with an Agilent 1260 series Infinity LC system (Agilent, Las Rozas, Madrid, Spain) with a quaternary pump, autosampler and column oven. The chromatography was performed at 30 °C with a Kromasil C18 column (250 × 50 mm, i.d. 4.6 µm) using a mobile phase composed of formic acid 0.1% (A) and acetonitrile (B). A gradient elution at a flow rate of 400 µL/min was applied: 0–5 min, 0% B; 5–8 min, 0–20% B; 8–11 min, 20–27% B; 11–13 min, 27–35% B; 13–20 min, 35–45% B; 20–23 min, 45–55% B; 23–28 min, 55–63% B; 28–32 min, 63–70% B; 32–37 min, 70–80% B, 37–40 min, 80% B; and returned to initial conditions in 5 min. The injection volume of the samples was 5 µL.

Electrospray ionization was performed in 4500 V negative and 5500 V positive mode. Parameters setting for temperature, curtain gas, ion source gas 1 and gas 2 were: 500 °C, 20 psi, 20 psi at flow 20 L/min. Data were acquired using the MRM (multiple reaction monitoring) mode. MRM mass spectrometry parameters DP (declustering potential), CXP (collision cell exit potential), CE (collision energy), EP (entrance potential) are summarized in Table S1 of Supplementary Materials. Chromatograms were integrated with MultiQuant software 1.0.3. (Sciex, Darmstadt, Germany).

Calibration curves were performed using the commercial standards, as previously described (Section 2.1. Chemicals and Reagents), in the range 1 µg/L–10 mg/L with the addition of 5 µL acetylsalicylic acid as an internal standard working solution (50 µg/L). Two sets of calibration curve samples were prepared on two different days. Individual signals were normalized, based on total weight, to account for sample variability and normalized peak areas for the internal standard.

All the samples were analyzed in three replicas intraday, and the analysis was repeated three times over a 6-month duration (interday). The limit of detection (LOD) and limit of quantification (LOQ) were used to determine the linearity, and all data were summarized in Table S2 of Supplementary Materials.

### 2.6. Statistical Analysis

The statistical analysis of the concentrations to determine the identified polyphenols was performed using SPSS [50] and R [51]. The descriptive statistics included: mean, median, mode and standard deviation. Shapiro–Wilk and Bartlett tests were performed to check the normality and homoscedasticity of data, respectively. Subsequently, ANOVA and post hoc Tukey (with Welch correction) tests were used to compare the quantity of polyphenols in different grape juices. Due to the high precision of the LC-MS/MS measurements, the obtained standard deviations were so small that a critical value of 0.01 was used to assess statistical significance.

The *p*-value results were combined with the fold of change—usually used in metabolomics [52]—to determine the functional relevance of concentration differences of polyphenols in the juice samples. The fold of change value is the ratio between the concentration of each polyphenol determined in the different grape juices, and the concentration in Airen grape juice, the latter of which was used as the reference. Functional relevance levels for the statistical tests were defined as *p*-values < 0.01, besides the fold of change values shown in Table 1. Levels 3 and 4 were determined as the ones with relevant concentration variations from the point of view of food and nutraceutical functionality, while levels 1 and 2 represent such small relative variations that they cannot be considered as relevant.

## 3. Results

### 3.1. Total Phenolic Content and Scavenging Activity of Extracts

The estimation of total polyphenols using spectrophotometric analysis determined that the highest concentration of compounds occurred in Tempranillo grape juice and its extracts (Table 2). When the white varieties were compared, Gewürztraminer grape juice had the highest polyphenol content, followed by Sauvignon Blanc, Airen and Verdejo grape juices. The estimated concentration of total polyphenols in Airen grape juice was similar to Sauvignon Blanc, 35% higher than the concentration estimated in Verdejo and 33% lower than the quantity detected in Gewürztraminer grape juice.

The estimated quantity of polyphenols detected in the extracts was lower than in the fresh grape juice, indicating a loss of polyphenols during the extraction process (Table 2). The loss of polyphenols varied according to the grape variety, estimated as 7.5% in Verdejo, 15% in Airen, 19.4% in Gewürztraminer, 24.7% in Sauvignon blanc, and 33.2% in Tempranillo. These differences could be attributed to the different polyphenol compositions of the grape juices. In fact, the red Tempranillo grape juice is known to be rich in proanthocyanidins and tannins, both being complex polyphenols that are poorly soluble in methanol. In the white grape juices, the high loss percentage determined in Sauvignon Blanc (24.7%) was surprising.

The antioxidant capacity of the grape juice and extracts studied was estimated using the DPPH method described in the Materials and Methods section. The highest DPPH scavenging activity (lower IC_50_ value) was detected in Tempranillo grape juice, followed by Gewürztraminer, Sauvignon Blanc, Airen and Verdejo (Table 2). The scavenging activity determined for the polyphenol extracts was lower (average reduction of 15%) in the white grape extracts, and lower by an average of 27% in the Tempranillo extract—a result consistent with the decrease in the concentration of total polyphenols (Table 2).

### 3.2. Identification and Quantification of Polyphenols by LC-MS/MS Analysis

The characterization of polyphenols in the grape juice extracts was performed by LC-MS/MS analysis. The separation of the compounds by LC was accomplished following the elution conditions described in the Materials and Methods section. For the quantification by MS, a database of 56 grape polyphenols with the MS parameters necessary for their identification was created using the data previously published [53,54,55,56,57,58,59,60,61,62,63,64,65,66,67] (Supplementary Materials, Table S3). Twenty-three of these polyphenols were selected for the study, and 15 have been identified in the extracts (Supplementary Materials, Table S2). These polyphenols belong to the following families: hydroxycinnamic acids (caffeic, chlorogenic and coumaric), hydroxybenzoic acids (dihydroxybenzoic, gallic, protocatechuic, salicylic and vanillic), stilbenes (resveratrol and polydatin), flavonoids (quercetin, isorhamnetin, catechin and epicatechin), and phenylpropanoids (esculetin). The quantification was performed with polyphenols without any chemical modification or isomerization.

#### 3.2.1. Polyphenols in Grape Juice Extracts

Three biological samples of each grape juice were analyzed by triplicate, and the mean concentration values obtained by LC-MS/MS were compared for each polyphenol in the different grape juice extracts. The extract of the Airen variety was used as the reference. We performed ANOVA and post hoc Tukey tests to determine if the observed differences among grape juices were statistically significant. In most cases, the tests resulted in statistically significant differences, even though the magnitude of the differences was consistently small. This can be explained in terms of the small standard deviations due to the high precision and the reproducibility of the LC-MS/MS technology used for the measurements (Table 3). The fold of change value was calculated for each polyphenol studied with respect to the Airen extract, and functional relevance was defined according to Table 1.

Reproducibility and variability were corroborated by the intraday experiments and by the experiments carried out on three more occasions over 6 months (interday). Completing the validation parameters, the LOD and LOQ of the analytical method were determined, limits which are not specific to the LC-MS/MS, but to the complete analytical method.

Three hydroxycinnamic acids were studied. Chlorogenic acid was detected in all five analyzed grape juice extracts. Tempranillo was the variety with the highest concentration and Sauvignon Blanc the one with the lowest quantity, both with a functional relevance level of 1 (Table 3). The two other acids analyzed were caffeic acid, detected in all the varieties except Sauvignon Blanc, and coumaric acid, which was only detected in the Airen and Verdejo extracts. The concentrations of these compounds in the extracts were very similar, and no functional relevance was determined.

Five hydroxybenzoic acids were studied. The detected concentrations of dihydroxybenzoic, protocatechuic, salicylic and vanillic acids were nearly identical in all extracts, with a functional relevance level of 1. Concentrations of gallic acid showed no statistical significance among the studied grape juices (Table 3).

With respect to the stilbenes examined, the concentrations of both resveratrol and polydatin were very similar across all grape varieties, although resveratrol was unexpectedly absent in the Sauvignon Blanc extract. In no case did the concentration differences observed in the extracts have a functional relevance (level 1).

**Figure 1:**
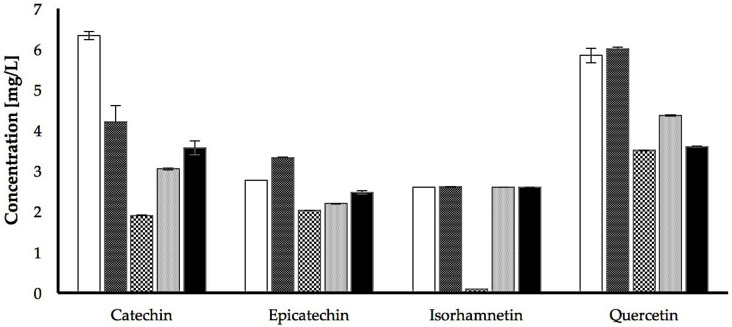
Concentrations of the flavonoids identified in the grape juice of *Vitis vinifera* varietals Airen (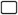), Gewürztraminer (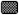), Sauvignon blanc (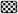), Verdejo (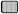), and Tempranillo (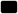).

The strongest differences were detected in the flavonoid family. It should be noticed that isorhamnetin was not detected in the Sauvignon Blanc extract, although concentrations in the other four grape juices were comparable (Table 3, Figure 1). Regarding epicatechin, the highest concentration was detected in Gewürztraminer, followed by Airen, Sauvignon Blanc being the grape juice with the least quantity (Table 3). The functional relevance value was 2 for all the varieties, with the exception of Tempranillo. In the case of quercetin, the highest concentration was found in the Airen and Gewürztraminer extracts, with lower concentrations for Verdejo (functional relevance level 2), and Sauvignon Blanc and Tempranillo (functional relevance level 3) (Table 3, Figure 1). Nevertheless, the greatest variation in concentration among the different extracts analyzed was detected for catechin. The highest concentration of catechin was discovered in the Airen extract, followed by Gewürztraminer, Tempranillo, Verdejo and Sauvignon Blanc. In fact, the differences in the concentrations showed a functional relevance level of 3 for all the varieties except Sauvignon Blanc, which had a functional relevance of 4 (Table 3, Figure 1).

Esculetin was the only polyphenol quantified from the phenylpropanoid family. This compound showed the lowest concentration in all the samples and the functional relevance value (level 1), indicating no relevant differences (Table 3).

**Figure 2:**
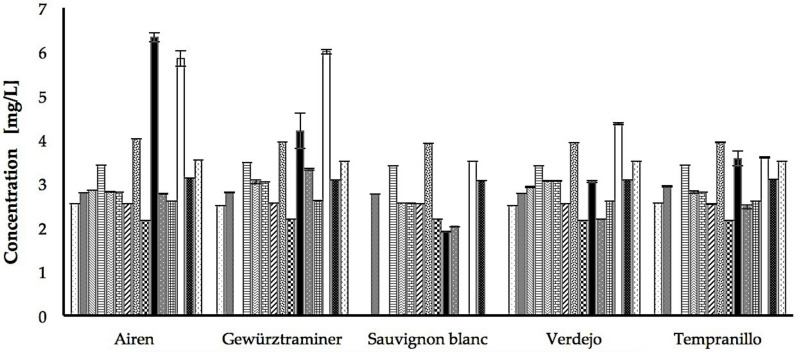
Comparison of the 15 polyphenols identified by LC-MS/MS in the grape juice extracts of *Vitis vinifera* var. Airen, Gewürztraminer, Sauvignon Blanc, Verdejo and Tempranillo. Caffeic acid (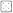), chlorogenic acid (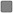), coumaric acid (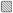), dihydroxibenzoic acid (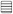), gallic acid (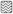), protocatechuic acid (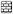), salicylic acid (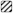), vanillic acid (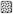), esculetin (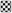), catechin (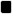), epicatechin (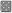), isorhamnetin (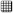), quercetin (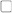), polydatin (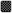), resveratrol (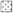).

Together, these results indicated that the global profiles of the 15 polyphenols analyzed in the Airen, Gewürztraminer, Sauvignon Blanc, Verdejo and Tempranillo grape juice extracts were very similar. However, the statistical analyses indicated that the majority (>90%) of the concentration differences detected in the samples were statistically significant; a result that, as previously explained, could be due to the precision and reproducibility of the technique used (LC-MS/MS). However, applying the fold of change criterion, only 17% of the statistically significant differences are considered to have functional relevance. This result is consistent with the qualitative analysis of the global polyphenol profile of the grape juice extracts shown in Figure 2, which clearly shows that only two polyphenols, quercetin and catechin, stand out in the Airen and Gewürztraminer grape juices above the others. The amount of quercetin in these two grape juices is very similar and is higher than the quantity detected in the rest of the grape juices (increases ranging between 25% and 65%). In the case of catechin, the highest concentration was found in the Airen samples, expressing levels 30% higher than the quantity detected in Gewürztraminer, and levels between 43% and 68% higher than the quantity detected in the other extracts.

#### 3.2.2. Effect of the Industrial Concentration Process on the Polyphenol Content of the Airen Grape Juice

The amount of total polyphenols estimated in the industrial samples revealed that the concentrated grape juice had fewer polyphenols than expected, specifically 42% and 44% lower than the initial fresh samples for the NCJ and DCJ samples, respectively (Table 4). These results indicated that the heating process for water evaporation negatively affects the stability of these compounds. Moreover, comparing the amount of total polyphenols estimated in the Airen grape juice samples before and after the ultrafiltration process resulted in a 17% reduction. The polyphenol extracts obtained from these samples were also compared, showing that in the case of the NCJ, the polyphenol losses oscillate between 9.6% and 5.9% in the initial and concentrated samples, respectively (Table 4). In the DCJ samples, polyphenol loss was higher, ranging between 15% and 33%, as determined for the initial and concentrated decolorized grape juices, respectively (Table 4). In summary, the results suggest that both the concentration and the ultrafiltration processes led to reductions in the amount of polyphenols in the concentrated final product.

The DPPH radical scavenging capacity of these samples was also determined. The obtained IC_50_ values showed that NCJ samples had more scavenging power than the decolorized samples (DCJ)—a finding that agrees with the higher amount of polyphenols and the absence of the ultrafiltration step in the NCJ samples, in comparison with DCJ (Table 4). Nevertheless, the putative antioxidant capacity of these samples was noteworthy, which led to the determination of how the concentration process affected the individual polyphenols previously identified by LC-MS/MS.

**Figure 3:**
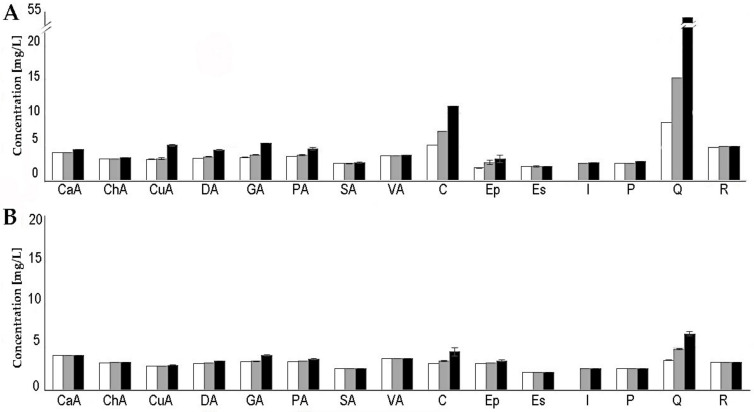
Concentration of the 15 polyphenols identified by LC-MS/MS in the Airen industrial grape juice samples at 19 °Bx (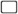), 30 °Bx (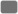) and 65 °Bx (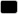). (**A**). Normal concentrated grape juice samples (NCJ). (**B**). Decolorized concentrated grape juice samples (DCJ). Abbreviations used correspond to caffeic acid (CaA), chlorogenic acid (ChA), coumaric acid (CuA), dihydroxybenzoic acid (DA), gallic acid (GA), protocatechuic acid (PA), salicylic acid (SA), vanillic acid (VA), catechin (C), epicatechin (Ep), esculetin (Es), isorhamnetin (I), polydatin (P), quercetin (Q) and resveratrol (R).

Eight phenolic acids were studied, and all were present in the final concentrated grape juice, although their concentration was much lower than expected considering the 3.5 times the sugars were concentrated. In the NCJ samples, the losses of these compounds ranged from 51% of coumaric acid to 70% of vanillic acid (Figure 3A, Supplementary Materials Table S4). In the DCJ samples, the losses ranged from 64% of gallic acid to 71% of vanillic acid (Figure 3B). The results were similar for the stilbene family, where losses of 68% and 71% for polydatin and resveratrol, respectively, were detected in the NCJ (Figure 3A, Supplementary Materials Table S4), and 71% for both compounds in the case of the DCJ samples (Figure 3B). The titration of esculetin revealed a loss of 70% and 71% in the NCJ and DCJ samples, respectively (Figure 3A,B, Supplementary Materials Table S4).

In the case of the flavonoids, the loss of these compounds was lower than that which was observed in previous cases, averaging 38% for catechin and 49% for epicatechin in the NCJ samples. The exception to this behavior was quercetin, which demonstrated an increase of 83% over the expected theoretical concentration in the NCJ samples (Figure 3A, Supplementary Materials Table S4). In the case of DCJ samples, losses were observed in all polyphenols of the family; 45% for quercetin, 58% for catechin and 68% for epicatechin (Figure 3B, Supplementary Materials Table S4). These results are consistent with the other polyphenols studied, except for the amount of quercetin detected in the NCJ sample.

The results indicate that the industrial process negatively affects the polyphenol content of the concentrated grape juice. In addition, losses are higher in DCJ samples compared to NCJ samples, which indicates that the filtration step of the grape juice prior to concentration is eliminating polyphenols from the grape juice. On the other hand, the unexpected increase of quercetin concentration in NCJ could be due to the presence of complex, mainly glycosylated molecules that, during the industrial heating of the grape juice, could be releasing free quercetin. This was not evident in the case of DCJ samples because the filtration process could retain these complex molecules; thus, the behavior of quercetin in these samples is similar to the rest of the analyzed polyphenols.

## 4. Discussion

The analysis of total polyphenols indicates that Tempranillo red juice has the highest concentrations, close to twice the concentrations detected in Airen and Sauvignon Blanc grape juices. Gewürztraminer is the white grape juice with the higher concentration of polyphenols, being 33% and 55% higher than the quantity detected in Airen/Sauvignon Blanc and Verdejo grape juices, respectively, but 31% lower than the concentration of these compounds in Tempranillo juice. The more precise analysis carried out by LC-MS/MS to determine the polyphenol profiles of 15 quantified compounds in grape juice extracts indicates that the polyphenols in the Airen variety are similar to the other white varieties analyzed. At this point, it should be noted that Airen and Gewürztraminer grape juices may demonstrate interesting differences in the concentration of the flavonoids, quercetin and catechin.

The results of this study showed that higher polyphenol concentration led to greater free-radical scavenging activity, a finding previously reported with these and other grape varieties [28,68]. In both grape juices and polyphenol extracts, Tempranillo has the highest antioxidant capacity, followed by Gewürztraminer, Sauvignon Blanc, Airen and Verdejo. This pattern follows that which is justified in the literature [30,69], but at the same time was surprising in relation to the Airen grape juice, which has always been considered a product of low quality. We found that the putative antioxidant capacity of Airen juice is not negligible, possibly due to the higher amount of quercetin and catechin compared to the other analyzed juices. In the Airen extract, 6.3 mg/L of catechin was detected, which is 33.3% more than the quantity present in Gewürztraminer (4.2 mg/L), 50% more than that detected in Verdejo (3.05 mg/L) and three times that of the concentration present in Sauvignon Blanc (1.91 mg/L). It also exceeds the catechin concentration detected in Tempranillo (3.6 mg/L) by 42%. Also noteworthy is the amount of quercetin detected in Airen (5.8 mg/L), which is similar to that of Gewürztraminer (6 mg/L) and considerably higher than that present in Sauvignon Blanc (3.5 mg/L) and Verdejo (4.37 mg/L) extracts. Quercetin detected in Tempranillo (3.6 mg/L) is very similar to that present in Sauvignon Blanc, and 40% lower than that detected in Airen. These results demonstrate the main differences in terms of polyphenol content in Airen grape juice, compared to the rest of the varietals analyzed. The differences in the concentrations recorded are statistically significant and have functional relevance. The quantification of epicatechin stands out to a lesser extent in Gewürztraminer (3.33 mg/L), which was 16.7% higher than that detected in Airen (2.77 mg/L), and more than 30% higher than that detected in the other three varieties: Sauvignon Blanc (2.03 mg/L), Verdejo (2.2 mg/L) and Tempranillo (2.48 mg/L). Although Tempranillo is a variety particularly rich in polyphenols [70,71], many of them have not been identified in this study, possibly because of the extraction process used. The polyphenols belonging to the anthocyanin and flavone families, which are responsible for the coloration of red varieties [28,68,69,72], contain more complex and apolar compounds that were not analyzed in this study.

The high concentration of quercetin and catechin highlighted in Airen grape juice is relevant because their anticarcinogenic properties have been shown to induce apoptotic pathways in cancer cells [73,74,75,76]. Many studies examining quercetin showed its effect in the modulation of signaling pathways and the expression of microRNAs directly involved in the development and progression of tumor cells [77,78,79]. In addition, the antioxidant activity neutralizing free ROS has been associated with the prevention of atherosclerosis [80] and cardiovascular diseases [17,81,82].

The results obtained with the industrial samples indicate that the concentration process of the Airen grape juice carried out by the company Mostos Españoles S.A. leads to a reduction in the total polyphenol content of between 38.4–69.6% and reaches 57.7–70% in the case of the discolored concentrated grape juice (DCJ). Data from the NCJ samples indicate that, as previously determined, the flavonoids catechin and quercetin stand out with respect to their high concentrations. Nevertheless, the concentration of most polyphenols in the final concentrated juice is lower than expected, in comparison with the quantity detected in the fresh juice, and the theoretical values calculated on the basis of the sugar concentration factor.

The case of quercetin is noteworthy because it is the only polyphenol that is not reduced during the concentration process. By contrast, it was unexpected that the concentration of this polyphenol in the concentrated sample NCJ_65_ is double that which would be expected if there were no loss of the compound. This could be explained by assuming that in the initial sample a significant part of the quercetin was glycosylated, and therefore would not have been adequately quantified by the method used. Glycosylation of flavonoids is common and is known to affect their solubility, stability and bioaccessibility [83]. If this is the case, the heat to which the grape juice is subjected during the concentration process could eliminate this modification and allow quantification of the compound in the final sample, because the ionization and fragmentation conditions of mass spectrometry are optimized to quantify free quercetin. This same phenomenon would also account for the loss of the remaining polyphenols during the concentration process. The heating to which the grape juice is subjected, in order for water evaporation to take place during concentration, is very likely to affect the structure of the polyphenols in a similar way to that which occurs when foods are cooked [84]. In some cases, these changes could lead to their degradation, modification and aggregation. This process is supported by the estimated 40% loss of total polyphenols in the concentrated grape juice, from 477 mg/L GAE in the initial grape juice to 927 mg/L in the NCJ_65_ (expected value 1669.5 mg/L). It is true that an increase in the free-radical scavenging activity of the concentrated grape juices was observed, from an IC_50_ value of 73 mg/L in the NCJ_19_ to 64 mg/L in the NCJ_65_, but in this case, it is challenging to make a comparison.

In the case of the DCJ samples, the estimated loss of total polyphenols associated with the concentration process is 56% and its antioxidant capacity decreases from an IC_50_ value of 50 mg/L in the DCJ_19_ to 31 mg/L in the DCJ_65_. Regarding the quantification of individual polyphenols in the DCJ by LC-MS/MS, the results are very similar to those obtained with the NCJ samples when a reduction in the concentration of all the polyphenols in the concentrated sample was detected. In this case, the loss of flavonoids is greater than in the NCJ_65_, but less than the losses recorded for the rest of the polyphenols studied. Losses of epicatechin, catechin, and quercetin are 67.8%, 57.7% and 44.9%, respectively. In this case, we cannot easily explain the reason why the loss of quercetin is similar to that detected in the rest of the polyphenols, and not the same as in the case of NCJ—where more catechin than theoretically predicted was detected. Fresh Airen grape juice is subjected to an ultrafiltration process prior to concentration for decolorization; this step could eliminate the catechin-derived molecules (glycosylated) that we have postulated as responsible for the high concentration of this polyphenol in the NCJ_65_ samples.

All these results suggest that the grape juice concentration process entails a loss of polyphenols, since the amount of these compounds in the concentrated grape juice is not proportional to the sugar concentration factor. It should be noted that the ultrafiltration process for obtaining the DCJ versus the NCJ includes a loss of total polyphenols, which translates into a difference ranging between 1% for phenolic acids and 18% for flavonoids. Furthermore, when comparing the amount of total polyphenols in the industrial juice samples before the concentration process (DCJ_19_ = 348.4 mg/L GAE and NCJ_19_ = 435 mg/L GAE) with that of the fresh Airen grape juice (638.86 mg/L GAE), an average decrease of 40% was observed. This data indicates that the storage conditions of the grape juice (90 days with sulfur dioxide at 880 ppm) negatively affect polyphenols, which is in agreement with other studies indicating that polyphenols degrade over time [85,86].

Our results show that concentrated grape juice, both normal (NCJ) and decolorized (DCJ), has a significant quantity of polyphenols, thus conveying the beneficial health properties associated with these compounds and the nutraceutical value when included in food stocks [3,34,87]. Both types of concentrated grape juice contain relevant amounts of quercetin and catechin, so by including this product in the formulation of any food, a small amount of these natural antioxidants is being added, contributing to the anti-inflammatory, anticarcinogenic, antimicrobial, anti-aging and cardioprotective associated effects. It is true that the bioavailability and pharmacokinetic properties of polyphenols depends on several factors, such as the food matrix and the concentration ingested, but there is evidence suggesting that dietary intake of polyphenols derived from grape juice has a positive effect on the gut microbiota and increases the amount of phenolic compounds in plasma [30,88]. Moreover, as previously mentioned, most of the grape polyphenols are present in the grape seeds and skin, so new procedures for grape juice obtention should be tested in order to increase the amount of these bioactive molecules in this product [28,89].

## 5. Conclusions

Our study provides compelling evidence that Airen grape juice has considerable amounts of polyphenols, with an outstanding concentration of the flavonols quercetin and catechin. This, in turn, supports the nutraceutical properties of this natural product and its use in the formulation of foods and drinks for children and athletes. The inclusion of grape juice in foodstuffs enriches them not only with natural sugars but also with polyphenols, bioactive molecules that promote health and prevent disease development, something that is directly linked to the sustainable development goal “Good Health and Well-being” of the UN 2030 agenda. However, additional research is needed to determine the content of other polyphenols, mainly polar compounds with higher solubility, that would contribute to extending the interest of this product in the Mediterranean diet.

## Figures and Tables

**Table 1:** Fold of change values associated with the defined functional relevance levels.

Fold of Change	[0.85–1); (1–1.15]	[0.4–0.7]; [1.3–1.6]	[0.4–0.7]; [1.3–1.6]	<0.4; >1.6
Functional relevance levels	1	2	3	4

**Table 2:** Total polyphenol content (TPC, expressed in mg/L GAE ± standard deviation) and DPPH scavenging activity (IC_50_, expressed in mg/L ± standard deviation) of the fresh juice and polyphenol extracts. All the comparisons between the Airen and the rest of varieties were statistically significant (*p*-value < 0.01), except for the Airen and Sauvignon Blanc TPC in fresh juice with a *p*-value = 0.0762 ^(a)^, the IC_50_ values in fresh juice, *p*-value = 0.99984 ^(b)^, and the polyphenol extract, *p*-value = 0.62657 ^(c)^.

	TPC (mg/L GAE)		IC_50_ (mg/L)	
*Vitis vinifera* Varieties	Fresh Juice	Polyphenol Extracts	Fresh Juice	Polyphenol Extracts
Airen	752 ± 47	639 ± 63	57 ± 5	67 ± 5
Gewürztraminer	1129 ± 6	910 ± 5	43 ± 6	49 ± 5
Sauvignon blanc	785 ± 66 ^(a)^	590 ± 35	57 ± 4 ^(b)^	64 ± 4 ^(c)^
Verdejo	491 ± 23	454 ± 11	67 ± 5	76 ± 5
Tempranillo	1639 ± 23	1094 ± 9	34 ± 4	46 ± 4

**Table 3:** Concentration of the 15 polyphenols identified in the grape juice extracts (mg/L ± standard deviation) including the relevance level of the determined differences. All *p*-values < 0.01 except those non-statistically different (nsd). nd (non-detected).

			Airen	Gewürztraminer	Sauvignon Blanc	Verdejo	Tempranillo
Hydroxycinnamic acids	Caffeic acid	Concentration (mg/L)	2.544 ± 0.002	2.5067 ± 0.0003	nd	2.4965 ± 0.0002	2.559 ± 0.002
		Relevance		1	--	1	1
	Chlorogenic acid	Concentration (mg/L)	2.791 ± 0.002	2.805 ± 0.003	2.765 ± 0.001	2.786 ± 0.002	2.94 ± 0.02
		Relevance		nsd (*p* = 0.0126)	1	nsd (*p* = 0.7823)	1
	Coumaric acid	Concentration (mg/L)	2.854 ± 0.001	nd	nd	2.927 ± 0.009	nd
		Relevance		--	--	1	--
Hydroxybenzoic acids	Dihydroxybenzoic acid	Concentration (mg/L)	3.422 ± 0.004	3.480 ± 0.002	3.404 ± 0.001	3.4114 ± 0.0003	3.423 ± 0.002
		Relevance		1	1	1	nsd (*p* = 0.624)
	Gallic acid	Concentration (mg/L)	2.81 ± 0.01	3.05 ± 0.04	2.559 ± 0.002	3.065 ± 0.009	2.81 ± 0.03
		Relevance		1	1	1	nsd (*p* = 0.998)
	Protocatechuic acid	Concentration (mg/L)	3.337 ± 0.002	3.338 ± 0.003	3.324 ± 0.001	3.333 ± 0.002	3.333 ± 0.003
		Relevance		1	1	1	1
	Salicylic acid	Concentration (mg/L)	2.5414 ± 0.0001	2.5621 ± 0.0003	2.5502 ± 0.0001	2.5412 ± 0.0001	2.5397 ± 0.0001
		Relevance		1	1	nsd (*p* = 0.0781)	1
	Vanillic acid	Concentration (mg/L)	4.024 ± 0.003	3.951 ± 0.005	3.9222 ± 0.0008	3.9323 ± 0.0005	3.943 ± 0.004
		Relevance		1	1	1	1
Estilbenes	Polydatin	Concentration (mg/L)	3.135 ± 0.003	3.0849 ± 0.0002	3.06735 ± 0.00002	3.0837 ± 0.0002	3.0989 ± 0.0007
		Relevance		1	1	1	1
	Resveratrol	Concentration (mg/L)	3.536 ± 0.002	3.512 ± 0.001	nd	3.511 ± 0.002	3.509 ± 0.0003
		Relevance		1	--	1	1
Flavonoids	Catechin	Concentration (mg/L)	6.3 ± 0.1	4.2 ± 0.4	1.910 ± 0.003	3.05 ± 0.02	3.6 ± 0.2
		Relevance		3	4	3	3
	Epicatechin	Concentration (mg/L)	2.772 ± 0.004	3.33 ± 0.02	2.02796 ± 0.00003	2.197 ± 0.005	2.48 ± 0.04
		Relevance		2	2	2	1
	Isorhamnetin	Concentration (mg/L)	2.6054 ± 0.0003	2.6137 ± 0.0006	nd	2.6029 ± 0.0002	2.6042 ± 0.0007
		Relevance		1	--	1	1
	Quercetin	Concentration (mg/L)	5.8 ± 0.2	6.00 ± 0.05	3.51 ± 0.01	4.37 ± 0.02	3.60 ± 0.01
		Relevance		nsd (*p* = 0.0264)	3	2	3
Phenylpropanoids	Esculetin	Concentration (mg/L)	2.171 ± 0.001	2.200 ± 0.001	2.196 ± 0.004	2.169 ± 0.001	2.164 ± 0.003
		Relevance		1	1	1	1

**Table 4:** Total polyphenol content (TPC, expressed in mg/L GAE ± standard deviation) and DPPH scavenging activity (IC_50_, expressed in mg/L ± standard deviation) of the industrial Airen samples and their extracts.

	TPC (mg/L GAE)		IC_50_ (mg/L)	
Airen Industrial Samples	Juice Samples	Polyphenol Extracts	Juice Samples	Polyphenol Extracts
NCJ_19_	477 ± 13	435 ± 15	73 ± 6	78 ± 7
NCJ_30_	649 ± 10	629 ± 51	62 ± 7	71 ± 7
NCJ_65_	927 ± 38	874 ± 14	64 ± 8	83 ± 7
DCJ_19_	490 ± 2	348 ± 31	50 ± 7	61 ± 8
DCJ_30_	643 ± 2	423 ± 12	52 ± 6	62 ± 8
DCJ_65_	769 ± 2	516 ± 13	31 ± 6	35 ± 7

## Supplementary Materials

The following are available online at https://www.mdpi.com/article/10.3390/foods10071532/s1. Table S1. Parameters to analyze the 23 selected polyphenols by mass spectrometry (10 volts were applied in all samples). Table S2. Calibration data of the polyphenols selected for their quantification and family to which they belong. Table S3. Selection of 56 polyphenols present in grapes with the most relevant parameters for their identification and analysis by mass spectrometry. Table S4. Concentration of the 15 polyphenols identified in the concentrated grape juice extracts (mg/L ± standard deviation) including the relative variation (%) between the determined concentration and the theoretical expected.
