# Supplementary material for: The High Content of Quercetin and Catechin in Airen Grape Juice Supports Its Application in Functional Food Production
Source: Foods. 2021 Jul 2;10(7):1532. doi: 10.3390/foods10071532 (PMC8306294; doi:10.3390/foods10071532)
Supplement: Supplementary file 1 [file foods-10-01532-s001.zip › foods-1231636-supplementary.pdf]

Supplementary Material

Table S1. Parameters to analyze the 23 selected polyphenols by mass spectrometry (10 volts were applied in all samples). Q1, m/z of the polyphenol to be analyzed. Q3, indicates the majority and stable fragment. CE (collision energy), DP (declustering potential), CXP (collision cell exit potential) and EP (entrance potential).

**Negative Ionization mode**							
**Nº**	**Compound name**	**Q1 (Da)**	**Q3 (Da)**	**Time (msec)**	**DP (V)**	**CE (V)**	**CXP (V)**
1	Caffeic acid	178.9	135	5	-50	-22	-9
2	Cumaric acid	162.9	93	5	-30	-30	-5
3	Dihydroxybenzoic acid	152.9	109	5	-75	-20	-7
4	Gallic acid	168.9	124.9	5	-110	-20	-7
5	Protocatechuic acid	152.9	90,9	50	-75	-32	-13
6	Salicylic acid	136.9	93,1	50	-15	-22	-5
7	Vanillic acid	167.1	152	50	-50	-20	-10
8	Esculetin	175.9	133	50	-65,5	-29	12
9	Isorhamnetin	315	300	50	-225	-28	-15
10	Polidatin	389.03	227.01	50	-110	-18	-9
11	Quercetin	301.1	178.9	50	-50	-21,15	-10
12	Resveratrol	226.98	142.89	50	-95	-30	-9
13	Rutin	609	299.9	50	-230	-70	-9
14	Viniferin	453	410.9	50	-45	-36,5	-9
**Positive Ionization mode**							
**Nº**	**Compound name**	**Q1 (Da)**	**Q3 (Da)**	**Time (msec)**	**DP (V)**	**CE (V)**	**CXP (V)**
15	Chlorogenic acid	355.1	163	50	46	21	10
16	Elagic acid	303.2	285.2	50	100	27	5
17	Ferulic acid	195	167	50	50	11.13	10
18	Apigenin	271	253.1	50	100	14	10
19	Catechin	291	139	50	16	21	10
20	Epicatechin	291	138.9	50	36	21	8
21	Kaempferol	285	153	50	111	43	10
22	Luteolin	285	153	50	100	89	10
23	Siringaldehyde	183	123.1	50	41	17	8

Table S2. Calibration data of the polyphenols selected for their quantification and family to which they belong. The correlation coefficient (r2), the limit of detection (LOD) and the limit of quantification (LOQ) are included for each of the polyphenols.

Family	Polyphenol	Ionization mode	Retention time (min)	Calibration curve	LOD (mg/L)	LOQ (mg/L)	r^2
Hydroxycinnamic acids	Caffeic acid	-	19.26	y = 244.62x – 604.68	0.228	0.759	0.994
	Chlorogenic acid	+	16.76	y = 334.98x - 917,07	0.022	0.075	0.996
	Cumaric acid	-	21.83	y = 5.1843x - 14.455	0.736	2.452	0.995
Hydroxycumarins	Esculetin	-	19.83	y = 17.313x - 37.133	0.288	0.961	0.999
Hydroxybenzoic acids	Protocatechuic acid	-	16.77	y = 1.332x - 4.3793	0.360	1.201	0.997
	Salicylic acid	-	28.33	y = 353.7x - 895.34	0.053	0.175	0.992
	Dihydroxybenzoic acid	-	16.78	y = 59.618x - 200.87	0.848	2.826	0.998
	Gallic acid	-	5.25	y = 20.642x - 50.802	0.895	2.983	0.989
	Vanillic acid	-	29.83	y = 4.6498x - 18.158	0.421	1.403	0.992
Flavonols	Isorhamnetin	-	31.84	y = 5.2125x - 13.553	1.128	3.760	0.997
	Quercetin	-	37.06	y = 1.2944x - 4.1921	1.221	4.070	0.998
Estilbenes	Polidatin	-	33.12	y = 39.529x - 89.901	0.715	2.383	0.997
	Resveratrol	-	26.35	y = 15.725x - 55.016	0.388	1.295	0.991
Flavanols	Catechin	+	133	y = 24.117x - 44.19	0.355	1.184	0.999
	Epicatechin	+	14.2	y = 18.621x - 37.673	0.877	2.925	0.997

Table S3. Selection of 56 polyphenols present in grapes with the most relevant parameters for their identification and analysis by mass spectrometry. Q1, (m/z) of the polyphenol to be analyzed. Q3, indicates the majority and stable fragment. CE is the collision energy. DP (declustering potential), CXP (collision cell output potential), EP (entrance potential). BR (bibliographic reference from which the information is derived).

Nº	Compound	Q1 (Da)	Q3(Da)	DP (V)	EP (V)	CE (V)	CXP (V)	BR
1	4-Metilumbeliferone	174.9	133.0	-135.0	-10.0	-28.0	-9.0	[53]
			119.1	-135.0	-10.0	-36.0	-7.0	
2	Aminobenzoic acid	137.1	77.0	56.0	10.0	29.0	10.0	
			94.0	56.0	10.0	19.0	8.0	
3	Benzoic acid	122.9	79.0	36.0	10.0	17.0	12.0	
			51.0	36.0	10.0	51.0	24.0	
4	Caffeic acid	178.9	135.0	-50.0	-10.0	-22.0	-9.0	
			107.0	-115.0	-10.0	-30.0	-7.0	
5	Caftaric acid	312.2	149.0	-100.0		-126.0	-10.0	[57]
6	Chlorogenic acid	355.1	163.0	46.0	10.0	21.0	10.0	[53]
			89.0	46.0	10.0	75.0	14.0	
7	Cumaric acid	162.9	119.0	-90.0	-10.0	-20.0	-7.0	
			93.0	-30.0	-10.0	-30.0	-5.0	
8	Dihydroxybenzoic acid	152.9	109.0	-75.0	-10.0	-20.0	-7.0	
			109.0	-75.0	-10.0	-20.0	-13.0	
9	Ellagic acid	303.2	285.2	100.0	10.0	27.0	5.0	[60]
			210.9	100.0	10.0	27.0	5.0	
			130.9	100.0	10.0	27.0	5.0	
10	Fertaric acid	325.1	193.0					[57]
11	Ferulic acid	195.0	176.9	21.0	10.0	11.0	10.0	[53]
			89.0	21.0	10.0	41.0	10.0	
12	Gallic acid	170.1	124.9	-110.0	-10.0	-20.0	-7.0	
			79.0	-110.0	-10.0	-30.0	-11.0	
13	Lipoic acid	205.1	171.0	-30.0	-4.0	-14.0	-1.0	[64]
			205.1	-25.0	-4.0	-5.0	-15.0	
14	Protocatechuic acid	152.9	90.9	-75.0	-10.0	-32.0	-13.0	[53]
16	Salicylic acid	136.9	93.1	-15.0	-10.0	-22.0	-5.0	
			64.9	-15.0	-10.0	-36.0	-11.0	
17	Sinapic acid	223.0	163.9	-120.0	-10.0	-20.0	-9.0	
			192.9	-120.0	-10.0	-28.0	-11.0	
18	Syringic acid	199.0	140.0	16.0	10.0	21.0	10.0	
			155.1	16.0	10.0	13.0	10.0	
19	Vanillic acid	166.9	137.0	-140.0	-10.0	-12.0	-9.0	
			109.1	-140.0	-10.0	-16.0	-7.0	
20	Apigenine	271.0	253.1	100.0	10.0	14.0	10.0	
			239.3	100.0	10.0	20.0	10.0	
21	Astilbin	451.1	305.1	147.1		85.0		[57]
22	Catechin	291.0	139.0	16.0	10.0	21.0	10.0	[53]
			123.0	16.0	10.0	19.0	8.0	
23	Chrysine	223.0	142.9	-170.0	-10.0	-36.0	-9.0	
			209.1	-170.0	-10.0	-30.0	-11.0	
24	Coniferaldehide	177.0	134.0	-30.0	-10.0	-28.0	-9.0	
			160.8	-30.0	-10.0	-28.0	-11.0	
25	Cumarin	165.1	147.0	150.0	10.0	65.0		[62]
26	Daidzein	255.2	199.0			35.0		[61]
27	Epicatechin	291.0	138.9	36.0	10.0	21.0	8.0	[53]
			123.0	36.0	10.0	21.0	8.0	
28	Epigallocatechin gallate	457.0	169.0	-165.0	-10.0	-20.0	-11.0	
			125.0	-165.0	-10.0	-52.0	-9.0	

29	Escopoletin	192.2	193.2	132.9	-10.0	-26.0	-11.0	[53]
30	Esculetin	175.9	133.1	-100.0	5.0	-25.0	-10.0	[63]
			105.0	-100.0	5.0	-21.0	-10.0	
31	Etil gallate	197.0	123.9	-135.0	-10.0	-20.0		[65]
32	Fisetin	285.1	134.9	-50.0	-7.0	-28.0	-15.0	[58]
33	Floretin	274.3	273.0	168.0	106.0			[67]
34	Floridzin	579.0	436.4					
35	Galangin	271.0	153.0	176.0	10.0	43.0	10.0	[53]
			115.1	176,0	10.0	59.0	8.0	
36	Hesperidin	611.2	303.0	51.0	10.0	31.0	16.0	
			153.1	51.0	10.0	67.0	10.0	
37	Isorhamnetin	315.0	300.0	-225.0	-10.0	-28.0	-15.0	
			150.9	-225.0	-10.0	-38.0	-9.0	
38	Kaempferol	285.0	229.0	-80.0	-10.0	-20.0		[55]
			255.0	-80.0	-10.0	-25.0		
		286.9	153.0	111.0	10.0	43.0	10.0	[53]
			268.9	100.0	10.0	17.0	9.0	
39	Luteolin	286.9	153.0	100.0	10.0	17.0	9.0	
			68.9	111.0	10.0	89.0	10.0	
40	Metil gallate	183.0	124.0	-98.0	-10.0	-30.0	-9.0	[56]
41	Myricetin	317.0	271.0	-75.0	-10.0	-20.0		[55]
			271.0	-75.0	-10.0	-20.0		
42	Naringenin	271.0	151.0	-130.0	-10.0	-24.0	-25.0	[53]
			119.0	-130.0	-10.0	-34.0	-11.0	
43	Naringin	579.0	271.0	-255.0	-10.0	-42.0	-13.0	
			151.0	-255.0	-10.0	-48.0	-9.0	
44	Pinobanksin	271.0	150.9	-140.0	-10.0	-24.0	-9.0	
			119.5	-140.0	-10.0	-32.0	-7.0	
45	Pinocembrin	256.3	153.0	96.0	10.0	33.0	10.0	
			76.9	96.0	10.0	73.0	10.0	
46	Propil gallate	211.0	123.9	-140.0	-10.0	-21.0		[65]
47	Pterostilbene	256.3		-50.0		-35.0		[66]
				43.0		35.0		
48	Pterostilbene dimer	511.2				35.0		
49	Pueraine	417.1	296.9	20.0	10.0	35.0	10.0	[61]
50	Quercetine	301.0	150.9	-50.0	-10.0	-28.0	-9.0	[53]
			121.0	-50.0	-10.0	-34.0	-7.0	
		301.0	151.0	-85.0	-10.0	-20.0		[55]
			271.0	-85.0	-10.0	-25.0		
		301.1	150.9	-50.0	-7.0	-28.0	-15.0	[58]
51	Rutin	609.0	299.9	-230.0	-10.0	-48.0	-15.0	[53]
			270.9	-230.0	-10.0	-70.0	-9.0	
52	Sinapaldehide	206.9	177.0	-20.0	-10.0	-26.0	-11.0	
			148.9	-20.0	-10.0	-34.0	-9.0	
53	Siringaldehide	183.0	123.1	41.0	10.0	17.0	8.0	
			77.0	41.0	10.0	31.0	10.0	
54	Taxifolin	304.3	259.1	-70.0	-10.0	-10.0	-3.0	[57]
		303.0	285.0	-70.0	-10.0	-10.0	-3.0	
55	*trans*-Resveratrol	228.2	91.0	75.0	10.0	32.0	7.0	
			135.0	75.0	10.0	21.0	10.0	
			107.0	75.0	10.0	32.0	8.0	
56	Viniferin	455.1	361.2					
			361.2					
		453.0	410.9	-45.0	-9.0	-36.5	-9.0	[59]

Table S4. Concentration of the 15 polyphenols identified in the concentrated grape juice extracts (mg/L ± standard deviation) including the relative variation (%) between the determined concentration and the theoretical expected. nd (non-detected).

			NCJ_{19}	NCJ_{30}	NCJ_{65}	DCJ_{19}	DCJ_{30}	DCJ_{65}
Hydroxycinnamic acids	Caffeic acid	Concentration (mg/L)	4.104±0.003	4.131±0.001	4.57±0.011	4.0127±0.0006	4.0294±0.0005	4.076±0.002
		% Variation		-36.26	-67.45		-36.4	-70.31
	Chlorogenic acid	Concentration (mg/L)	3.2203±0.0004	3.236±0.001	3.361±0.007	3.201±0.001	3.2049±0.0002	3.232±0.006
		% Variation		-36.36	-69.49		-36.59	-70.49
	Cumaric acid	Concentration (mg/L)	3.14±0.02	3.24±0.11	5.23±0.14	2.81±0.02	2.810±0.008	2.91±0.02
		% Variation		-34.65	-51.32		-36.64	-69.76
Hydroxybenzoic acids	Dihydroxybenzoic acid	Concentration (mg/L)	3.296±0.012	3.497±0.036	4.496±0.088	3.08±0.01	3.142±0.006	3.418±0.005
		% Variation		-36.67	-62.4		-35.28	-67.51
	Gallic acid	Concentration (mg/L)	3.41±0.01	3.796±0.071	5.5±0.2	3.30±0.02	3.36±0.02	4.05±0.105
		% Variation		-29.49	-52.56		-35.56	-64.19
	Protocatechuic acid	Concentration (mg/L)	3.55±0.01	3.75±0.04	4.7±0.1	3.34±0.01	3.407±0.004	3.59±0.06
		% Variation		-33.18	-61.4		-35.36	-68.6
	Salicylic acid	Concentration (mg/L)	2.523±0.001	2.526±0.002	2.621±0.006	2.511±0.001	2.511±0.001	2.5271±0.0004
		% Variation		-36.6	-69.64		-36.67	-70.58
	Vanillic acid	Concentration (mg/L)	3.663±0.001	3.665±0.001	3.717±0.001	3.6583±0.0005	3.6582±0.0006	3.6643±0.0008
		% Variation		-36.59	-70.34		-36.67	-70.72
Estilbenes	Polidatin	Concentration (mg/L)	2.531±0.002	2.55±0.01	2.80±0.03	2.5016±0.0001	2.5002±0.00001	2.509±0.0001
		% Variation		-36.09	-67.66		-36.66	-70.69
	Resveratrol	Concentration (mg/L)	4.893±0.002	5.07±0.03	5.083±0.006	3.2404±0.0002	3.2404±0.0003	3.2415±0.0003
		% Variation		-34.34	-69.63		-36.67	-70.76
Flavonoids	Catechin	Concentration (mg/L)	5.26±0.09	7.4±0.4	11.1±0.5	3.081±0.007	3.34±0.07	4.5±0.5
		% Variation		-11.47	-38.37		-30.12	-57.74
	Epicatechin	Concentration (mg/L)	1.84±0.01	2.7±0.1	3.20±0.03	3.089±0.002	3.149±0.008	3.399±0.11
		% Variation		-7.86	-49.26		-35.42	-67.82
	Isorhamnetin	Concentration (mg/L)	nd	2.528±0.002	2.613±0.006	nd	2.5125±0.0004	2.5155±0.0007
		% Variation		nd	nd		nd	nd
	Quercetin	Concentration (mg/L)	8.7±0.3	15.30±1.03	54.3±1.3	3.48±0.02	4.797±0.02	6.6±0.25
		% Variation		+11.81	+83.12		-12.66	-44.9
Phenylpropanoids	Esculetin	Concentration (mg/L)	2.054±0.001	2.057±0.001	2.078±0.001	2.052±0.001	2.053±0.001	2.054±0.001
		% Variation		-36.6	-69.51		-37.7	-70.02
